# Movement of cerebrospinal fluid tracer into brain parenchyma and outflow to nasal mucosa is reduced at 24 h but not 2 weeks post-stroke in mice

**DOI:** 10.1186/s12987-023-00427-2

**Published:** 2023-04-11

**Authors:** K. E. Warren, K. G. Coupland, R. J. Hood, L. Kang, F. R. Walker, N. J. Spratt

**Affiliations:** 1 School of Biomedical Sciences and Pharmacy, University of Newcastle, Callaghan and Hunter Medical Research Institute, University Drive, Callaghan, New Lambton Heights, NSW 2308 Australia; 2 Hunter New England Health District, New Lambton Heights, NSW Australia

**Keywords:** Stroke, Ischaemic stroke, Cerebrospinal fluid, Intracranial pressure, Cribriform plate

## Abstract

**Background:**

Recent data indicates that cerebrospinal fluid (CSF) dynamics are disturbed after stroke. Our lab has previously shown that intracranial pressure rises dramatically 24 h after experimental stroke and that this reduces blood flow to ischaemic tissue. CSF outflow resistance is increased at this time point. We hypothesised that reduced transit of CSF through brain parenchyma and reduced outflow of CSF via the cribriform plate at 24 h after stroke may contribute to the previously identified post-stroke intracranial pressure elevation.

**Methods:**

Using a photothrombotic permanent occlusion model of stroke in C57BL/6 adult male mice, we examined the movement of an intracisternally infused 0.5% Texas Red dextran throughout the brain and measured tracer efflux into the nasal mucosa via the cribriform plate at 24 h or two weeks after stroke. Brain tissue and nasal mucosa were collected ex vivo and imaged using fluorescent microscopy to determine the change in CSF tracer intensity in these tissues.

**Results:**

At 24 h after stroke, we found that CSF tracer load was significantly reduced in brain tissue from stroke animals in both the ipsilateral and contralateral hemispheres when compared to sham. CSF tracer load was also reduced in the lateral region of the ipsilateral hemisphere when compared to the contralateral hemisphere in stroke brains. In addition, we identified an 81% reduction in CSF tracer load in the nasal mucosa in stroke animals compared to sham. These alterations to the movement of CSF-borne tracer were not present at two weeks after stroke.

**Conclusions:**

Our data indicates that influx of CSF into the brain tissue and efflux via the cribriform plate are reduced 24 h after stroke. This may contribute to reported increases in intracranial pressure at 24 h after stroke and thus worsen stroke outcomes.

**Supplementary Information:**

The online version contains supplementary material available at 10.1186/s12987-023-00427-2.

## Background

Disruptions to cerebrospinal fluid (CSF) dynamics contribute to the pathogenesis of several neurological conditions including subarachnoid haemorrhage, traumatic brain injury, and Alzheimer’s and Parkinson’s diseases [1]. Exchange between CSF and interstitial fluid in the parenchyma, via perivascular spaces or other routes, such as movement across the pia mater, appears to be responsible for ‘rinsing’ waste from the brain [2] and facilitating the movement of molecules necessary for central nervous system homeostasis [3–6]. This system has been described in detail in a review by Hladky and Barrand (2014) [7]. Disruption to CSF circulation is associated with deposition of neurotoxic proteins, such as amyloid-β [2], that contribute to neurological and neurodegenerative disease.

Ischaemic stroke is another neurological condition which disrupts CSF dynamics [8, 9]. More than one study has reported reduced CSF movement within the parenchyma after stroke and this has been found to correspond with decreased clearance of focal solutes [9] which could explain the increased amyloid-β deposition and reduced cognitive function identified by others [10, 11]. These ‘classical’ hallmarks of CSF dysregulation may not be the only way that altered CSF dynamics contribute to stroke sequelae. Dysfunctional CSF dynamics are known to contribute to conditions where intracranial pressure (ICP) becomes elevated, such as hydrocephalus [12]. We have previously identified a transient elevation in ICP in the first 24 h after stroke in rats [13]. This post-stroke increase in ICP reduces blood flow in collateral vessels which would feasibly lead to infarct expansion [14]. Further investigation of this phenomenon by our team and others indicated that increased CSF outflow resistance [15] and reduced clearance to cervical lymphatics contributes to this ICP rise [16]. Bothwell et al. (2021) reported reduced CSF efflux to the deep cervical lymph nodes after stroke [16] but what remains unclear is which particular routes of CSF outflow from the craniospinal space are disturbed after stroke and whether this decrease in outflow persists beyond the acute phase of stroke. Furthermore, it remains to be determined whether reduced CSF movement within the cranial space also occurs when ICP elevation peaks after stroke.

CSF exits the central nervous system via three main routes: cranial and spinal arachnoid granulations within the dural venous sinuses, perivascular pathways and meningeal lymphatics, and outflow along cranial nerves to exit sites such as the cribriform plate [17, 18]. The cribriform plate in particular has been demonstrated to be an important site of CSF outflow in many species [19, 20]. It is feasible to hypothesise that a reduction in CSF circulation in the parenchyma after stroke could correspond with reduced CSF outflow, but as yet no one has investigated whether disturbances in CSF circulation and CSF outflow are temporally aligned, and whether these disturbances line up with ICP elevation peaks after stroke.

We hypothesised that reduced penetration of CSF into the brain parenchyma and reduced outflow of CSF via the cribriform plate would be present 24 h after stroke, the time at which post-stroke ICP elevation peaks in rats. Using the same mouse model of stroke as outlined in this paper, we identified a similar ICP rise 24 h after stroke, indicating that the same phenomenon is occurring in our current model (unpublished data). We also wished to determine whether disruption to CSF dynamics persisted beyond the acute phase when ICP is no longer elevated. Therefore, the aim of this study was to assess CSF movement in the brain and outflow via the cribriform plate at 24 h and two weeks post-stroke.

## Methods

### Animals

Adult male 10–14 week old C57BL/6 mice weighing 25–32 g were used for this study (n = 35; Animal Services Unit, University of Newcastle). Animals were housed under standard conditions with a 12-hour light-dark cycle and unlimited access to food and water. Animals were excluded from the study if any of the following occurred: premature death, incomplete paraformaldehyde perfusion, damage to tissue sections, or if there was no evidence of stroke post-mortem (stroke group).

### Experimental design

Animal research was undertaken in accordance with the Animal Research: Reporting of In Vivo Experiments (ARRIVE) guidelines [21]. Mice were randomised to stroke or sham groups and 24 h or 2-week groups prior to stroke induction. The photothrombotic stroke model was chosen for stroke induction as it produces small, consistent lesion volumes with little edema. The 24 h and 2-week time points were selected to determine whether CSF dynamics and outflow are disrupted at a time when ICP has been shown to peak after stroke (24 h), and whether this disruption of CSF dynamics persists beyond the acute phase of stroke (two weeks). Tracer was allowed to circulate for 30 min based on a pilot time course study that indicated maximal dye penetration into the parenchyma at 30-minutes (Supplementary Fig. 1). This part of the experiment was performed under ketamine/xylazine anesthesia in order to maintain the CSF system as physiologically normal as possible under general anesthetic [22]. Analysis of CSF tracer in nasal mucosa was performed blinded, however it was not possible to blind analysis of brain tissue due to the presence or absence of a lesion.

### Anaesthesia and monitoring

For stroke induction, mice were anaesthetised with 5% isoflurane in 100% oxygen in an induction chamber. Anaesthesia was maintained at 1.5–2.5% isoflurane, in 100% oxygen. For cisterna magna injection, mice were briefly inducted on 5% isoflurane in 100% medical air in an induction chamber, then maintained under intraperitoneal 50–100 mg/kg ketamine and 5–10 mg/kg xylazine in 100% medical air. Body temperature was continuously monitored throughout surgery via a rectal probe coupled to a thermoregulatory heat mat (RET-2, Physitemp Instruments Inc., U.S.A.). Incision sites were shaved, cleaned, and injected subcutaneously with 2 mg/kg 0.05% Bupivacaine. After stroke surgery, animals received 1.5 mL of subcutaneous 0.9% saline to prevent dehydration and were returned to their home cage with free access to softened chow and water.

### Photothrombotic stroke

Photothrombotic stroke was induced in the somatosensory/motor cortex of mice as outlined previously [23]. The white light photothrombotic model was used for this study given its production of a consistent, focal lesion that does not affect large arteries. As a result, changes in blood flow or vessel pulsatility are not expected to be different in tissue outside of the area of illumination. This has been demonstrated by Arbel-Ornath et al. 2013 who noted no change in red blood cell velocity in a non-occluded vessel outside the area of illumination [24].

Mice were given an intraperitoneal injection of 0.2 mL of 10 mg/mL Rose Bengal (Sigma Aldrich, U.S.A) in 0.9% saline for stroke animals or saline only for sham. The scalp was shaved, cleaned, and a midline incision made to expose the skull. Scalp was retracted to expose skull overlaying the somatosensory cortex. Following 10 min of Rose Bengal circulation, a cold light source (Olympus, Japan) was placed over the skull 2.2 mm lateral to Bregma and the skull illuminated for 15 min. Following illumination, the scalp incision was closed using Vetbond (3 M, U.S.A.).

### Administration of fluorescent tracer

At 23.5 hours (n = 14) or two weeks (n = 19) post-stroke, mice received an intracisternal injection of 0.5% Texas Red dextran (3 kDa; Invitrogen, Thermo Fisher Scientific, U.S.A.) in freshly prepared artificial cerebrospinal fluid (NaCl: 119.4mM; Glucose: 11mM; NaHCO_3_: 26mM; KCl: 2.5mM; NaH_2_PO_2_: 1mM; MgCl_2_: 1mM; CaCl_2_: 2.5mM, pH 7.4). For injection of the tracer, mice were positioned prone with the head tucked toward the sternum at a 135° angle. Following injection of local anaesthetic as above, an incision was made from the occipital crest to the nape of the neck and the 3 layers of muscle carefully separated using curved forceps to expose the dura mater. A custom cannula comprising a 15 cm piece of PE-20 tubing (0.015” ID; S.A.I. Infusion technologies, U.S.A.) and a 30 G needle (BD, U.S.A), preloaded with tracer, was inserted 2 mm into the cisterna magna and kept in place using Vetbond (3 M, U.S.A.). Using a 10 µL Hamilton syringe and syringe pump (Stoelting, Germany), tracer was injected at 1 µL/min for a total of 10 min. This infusion has been shown to produce a transient increase in ICP that normalises within 5 min of cessation of tracer infusion [6, 25]. At the end of injection, the cannula was cut to ~ 3 cm in length and sealed with cyanoacrylate (UHU, Germany) and glue accelerator (ZAP, U.S.A.) to prevent backflow of tracer and CSF. The head was then placed flat to allow for physiological CSF flow and the mouse maintained under anaesthesia on a heat mat at 37 °C for 30 min while the tracer circulated [26].

### Tissue processing

Mice were overdosed with sodium pentobarbital (ProVet, Australia) 30 min after the start of tracer infusion and transcardially perfused with 0.9% saline until exsanguinated followed by 4% paraformaldehyde to fix tissue. After transcardial perfusion, mice were decapitated, and skin and muscle were cut away from the skull using scissors and the mandible, tympanic bulla, and zygomatic arches removed. The dorsal skull cap was removed, and the brain extracted and post-fixed in a 4% paraformaldehyde/12.5% sucrose solution for 4 h, followed by 12.5% sucrose solution until sectioning. The ventral skull was post-fixed in a 2% paraformaldehyde solution for 2 h at 4 °C. Skulls were then decalcified by incubating in 0.5 M EDTA (pH 7.4) for 24 h at 4 °C. The nasal region was separated from the ventral skull by cutting along the maxillary suture and mounted in Tissue-TEK O.C.T. (Sakura Finetek, U.S.A.) using truncated pyramid Peel-A-Way moulds (Polysciences, U.S.A.) and stored at -80 °C until sectioning.

Brains were sectioned into 60 μm coronal sections within 24 h of perfusion fixation, using a freezing microtome. Sections were immediately mounted onto gelatin coated slides and coverslipped using Vectashield anti-fade medium with 4′,6-diamidino-2-phenylindole (DAPI; Vectorlabs, U.S.A.). The nasal region was sectioned into 200 μm coronal sections using a cryostat at -15 °C to produce 4 coronal tissue levels (level 1 being the most proximal to the cribriform plate; Fig. 4a). These sections were washed with PBS to remove Tissue-TEK O.C.T. and mounted with Vectashield anti-fade mounting medium (Vectorlabs, U.S.A.) and 0.2 mm mounting spacers (SUNJin Lab, China).

### Fluorescent imaging

**Fig. 1:**
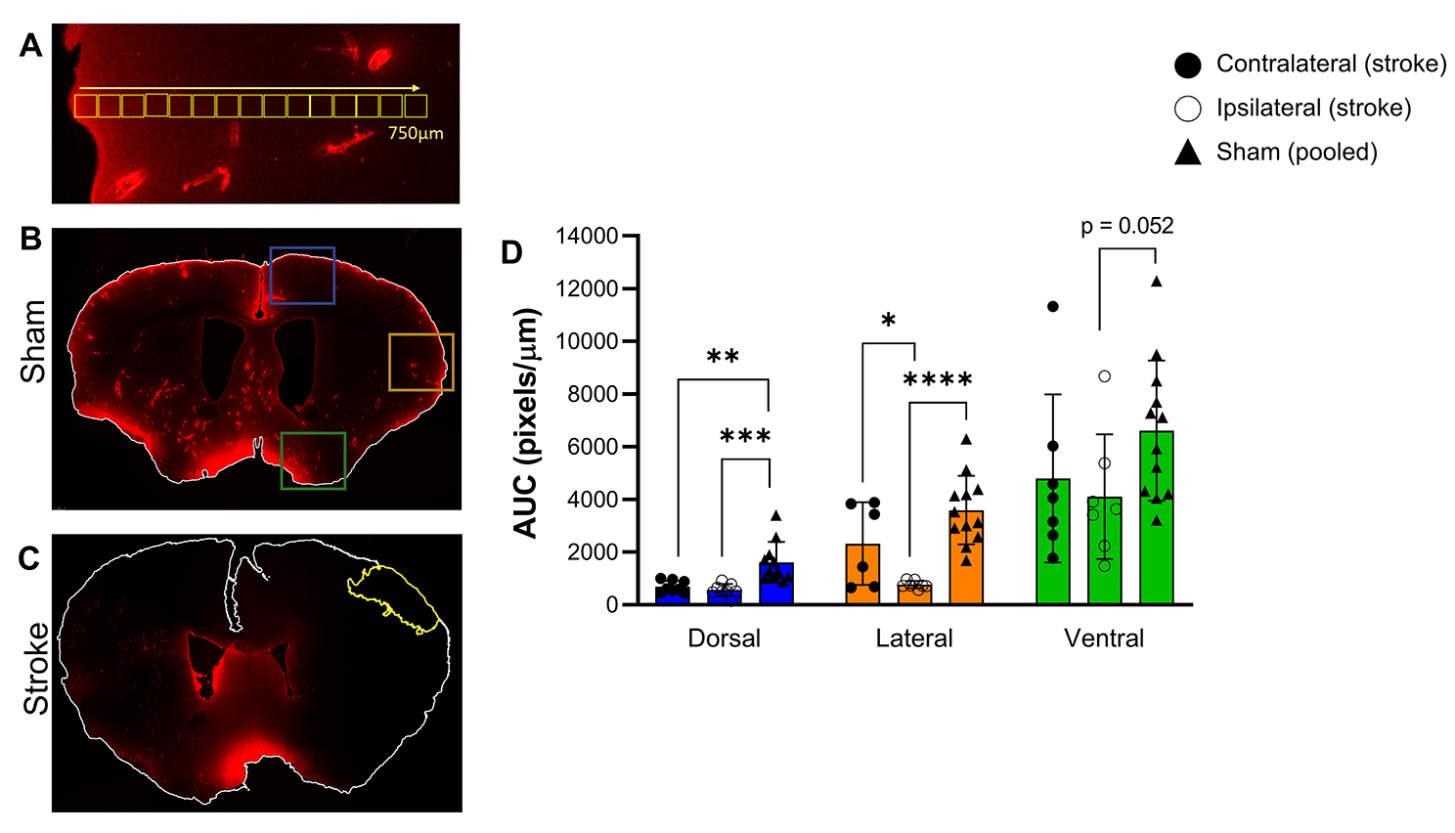
CSF tracer distribution 24 h after photothrombotic stroke. (A) Dye penetration into parenchyma was assessed by quantifying fluorescent parenchymal signal in 15 sequential 50 µm^2^ box regions of interest (ROI) from the pial surface to 750 µm^2^ depth. These ROIs were contained within the region of the coloured boxes shown in B. (B) Representative maximal projection of coronal section from a sham animal indicating the dorsal (blue), lateral (orange), and ventral (green) regions used to quantitate CSF tracer influx. (C) Representative maximal projection of coronal section from a photothrombotic stroke animal. The infarct is outlined in yellow. (D) The mean pixel intensity in the dorsal, lateral, and ventral regions of sham, and stroke ipsilateral and contralateral hemispheres plotted as mean fluorescent pixels/µm from brain surface to give the area under the curve (AUC). **** p < 0.005, *** p < 0.001, **p < 0.01, *p < 0.05. Exposure of the representative images (B and C) was increased for publication.

Imaging was performed using a Lecia TCS SP8 confocal microscope with a Leica HC PLC APO 10 x/0.40 mm objective. For brain, a dorsal, lateral, and ventral region at level Bregma 0.2 mm was imaged as a z-stack (2 μm step size) in both the contra- and ipsilateral hemispheres (Fig. 1b, sham; Fig. 1c, stroke). Bregma level for final analysis was chosen by comparing tracer load across multiple levels in two animals. Tracer load was comparable across all levels examined. For the nasal region, a z-stack tile scan of the entire section (5 μm step size) was taken of each of the levels under both brightfield and the 637 nm laser. Imaging parameters (laser power, spectrum wavelength, resolution, and gain) were held constant throughout all imaging sessions.

### Image analysis

All analyses were performed using maximal projections of z-stacks in ImageJ. CSF tracer load in the dorsal, lateral, and ventral regions was examined *ex vivo* to determine whether differences in tracer influx were altered close to the stroke as well as at sites distal to the stroke. For quantitation of fluorescent tracer in *ex vivo* brain tissue we adapted a method from Howe et al.,[10]. Briefly, sequential 15 × 50 µm^2^ regions of interest (ROIs) starting at the brain surface to 750 μm deep were placed over the maximum projection image of each brain region (i.e., lateral, ventral or dorsal; Fig. 1a). The boxes were placed to capture only parenchymal signal. Perivascular signal and infarct tissue was specifically avoided. Mean pixel intensity was recorded in each ROI and this was repeated three times per region, per brain. Mean pixel intensity was taken as an average of the triplicates. This mean pixel intensity was divided by the mean pixel intensity from mice injected with aCSF (i.e., no fluorescent tracer) to normalise for background fluorescence. These values were plotted as mean pixel intensity relative to distance from brain surface for each region. From this plot, area under the curve (AUC) was calculated with a greater AUC corresponding to greater tracer load. For nasal mucosa, the ImageJ thresholding tool was used to determine first, the total fluorescent area for confocal images, and second, total tissue area for brightfield images. Thresholding was performed by two independent, blinded investigators. Final values are an average of the values from the two investigators. Brightfield images provided the total area of nasal mucosa and laser fluorescence images provided the area of the nasal mucosa containing fluorescent tracer. Relative quantitation of fluorescent tracer was calculated as a percent of the total nasal region imaged.

### Statistics

Statistical analysis was performed with GraphPad Prism v9 software (GraphPad, U.S.A). Data was assessed for normality (Shapiro-Wilk) and the appropriate statistical test chosen for each data set. Paired t-tests were used for normally distributed data or Wilcoxon tests for non-normally distributed data to compare differences between the left and right hemispheres of stroke and sham animals in the three brain regions (dorsal, lateral, and ventral). If there were no significant differences in regions across hemispheres for sham animals, the values for each ipsilateral and contralateral region were pooled. These pooled sham values were then compared to corresponding brain regions of stroke animals using unpaired t-tests with Welch’s correction for normally distributed data or Mann-Whitney tests for non-normally distributed data.

For nasal tissue, comparisons were made between sham and stroke for each tissue level using unpaired t-tests with Welch’s correction. Significance was accepted at p < 0.05. Data is presented as mean ± standard deviation (SD).

## Results

### Inclusions/exclusions

In the 24-hour post-stroke cohort, brain tissue from six sham and eight stroke mice were included. Nine animals were excluded from analysis due to premature death (n = 6), incomplete paraformaldehyde perfusion (n = 1) and failed stroke (n = 2). In the two-weeks post-stroke cohort, brain tissue from eight sham and 12 stroke mice were included. Eight animals were excluded from analysis due to premature death (n = 4) and failed stroke (n = 4). Processing nasal mucosa for imaging is technically challenging and resulted in some sections being damaged. Damaged sections were excluded from final imaging. As a result, nasal mucosa tissue was taken from six stroke and six sham mice at 24 h and six stroke and four sham mice at two weeks post-stroke.

### CSF tracer load in brain parenchyma at 24 h post stroke

Average lesion volume was 7.6 ± 2.14 mm^3^ at 24 h after stroke, and 3.35 ± 1.32 mm^3^ at two weeks after stroke.

We first examined CSF tracer load in the ipsilateral hemisphere compared to the contralateral hemisphere in stroke animals at 24 h post-stroke. The lateral region exhibited a significantly reduced amount of tracer in the ipsilateral hemisphere (765 ± 142 pixels/µm) compared to the contralateral hemisphere (2583 ± 1588 pixels/µm, p = 0.031; Fig. 1d). Tracer load was comparable between ipsi- and contralateral hemispheres in the dorsal and ventral regions.

Next, we examined CSF tracer load in the dorsal, lateral and ventral regions of the brain compared to their sham counterparts. No significant difference between hemispheres were seen for shams, so values were pooled. There was a marked decrease in the amount of CSF tracer present in the ipsilateral hemisphere of stroke animals in both the dorsal (stroke = 561 ± 231 pixels/µm, sham = 1631 ± 743, p = 0.0004) and lateral (stroke = 765 ± 142 pixels/µm, sham = 3592 ± 1299 pixels/µm, p < 0.0001) regions, and a trend towards a decrease in the ventral region, compared to shams (stroke = 4103 ± 2369 pixels/µm, sham = 6608 ± 2658 pixels/µm, p = 0.052; Fig. 1d). Interestingly, in the contralateral hemisphere there was also a significant decrease in the amount of CSF tracer present in the dorsal region compared to shams (stroke = 681.6 ± 227 pixels/µm, sham = 1631 ± 743 pixels/µm, p = 0.001; Fig. 1d), but no significant differences were seen in the lateral and ventral regions.

### CSF tracer load in brain parenchyma at 2 weeks post stroke

**Fig. 2:**
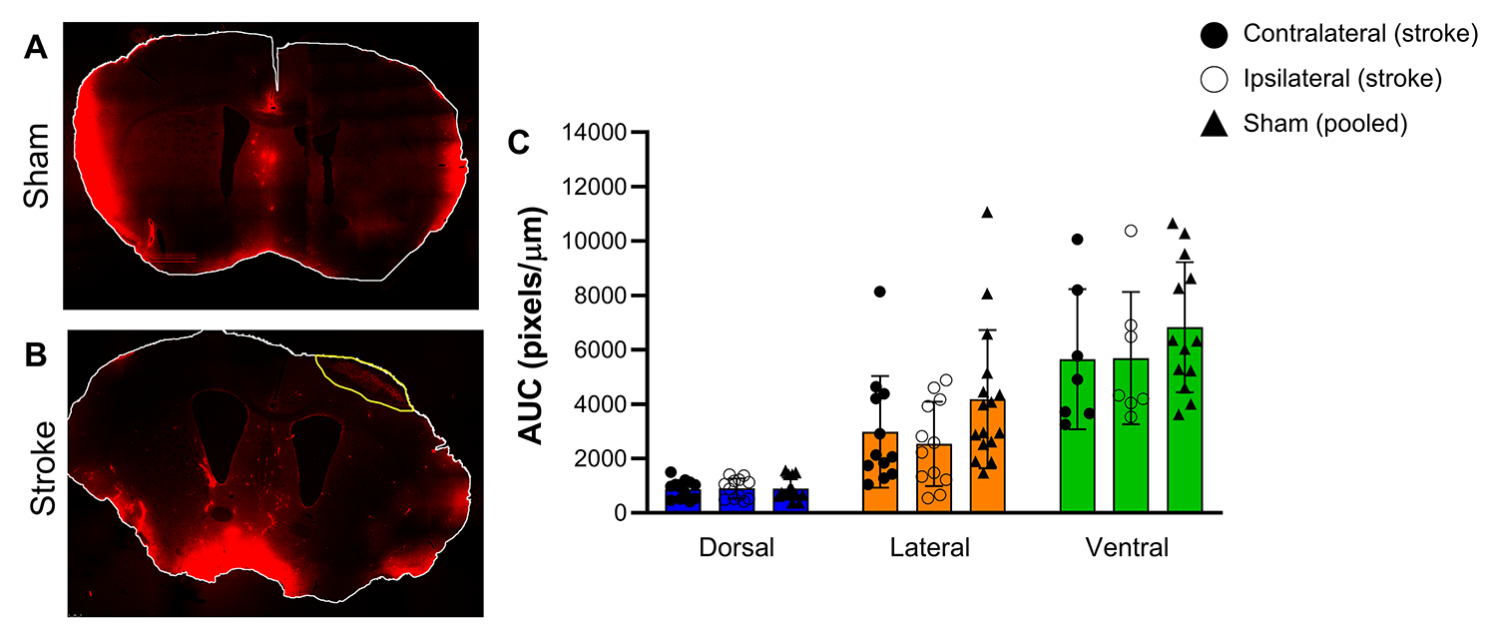
CSF tracer distribution two weeks after photothrombotic stroke. (A) Representative maximal projection of coronal section from a sham animal. (B) Representative maximal projection of coronal section from a photothrombotic stroke animal. The infarct is outlined in yellow. (C) The mean pixel intensity in the dorsal, lateral, and ventral regions of sham and stroke ipsilateral and contralateral hemispheres plotted as mean fluorescent pixels/µm from brain surface to give the area under the curve (AUC). Exposure of the representative images (A and B) was increased for publication.

At two weeks post stroke, there were no significant differences in CSF tracer load between the ipsilateral and contralateral hemispheres of stroke animals for any of the regions examined (Fig. 2c). In addition, there was no difference in CSF tracer load between ipsilateral or contralateral regions compared to sham animals (Fig. 2c).

### CSF tracer load in different brain regions

**Fig. 3:**
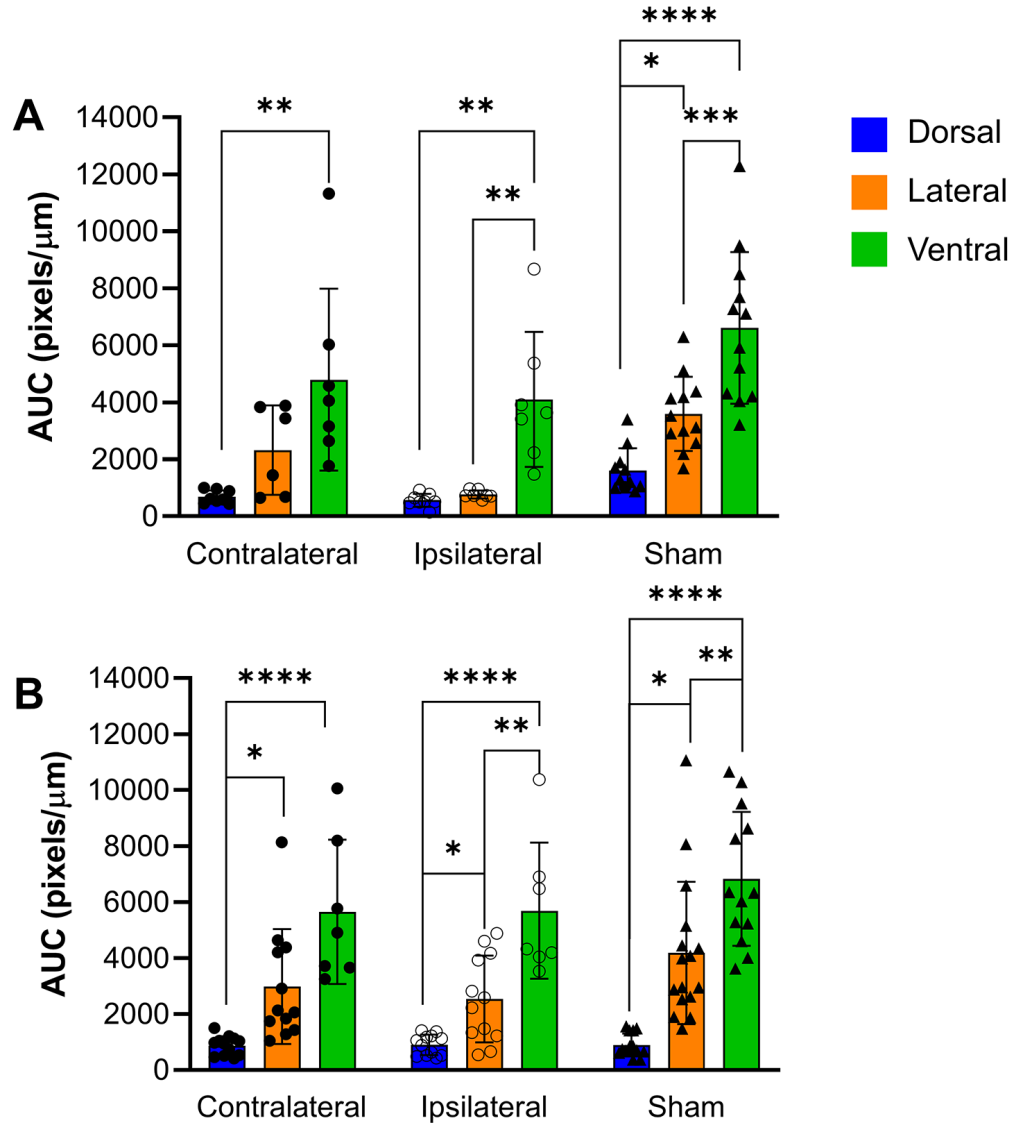
Comparison of CSF tracer load between the dorsal, lateral and ventral regions of the brain at (A) 24 h, and (B) two weeks post-stroke.

Differences in tracer load were noted in each of the brain regions examined. In sham animals, there was significantly more tracer in the lateral and ventral regions compared to the dorsal region at both the 24 h and two-week time point (Fig. 3). There was also significantly higher tracer load in the ventral region compared to the lateral region at both time points.

**Fig. 4:**
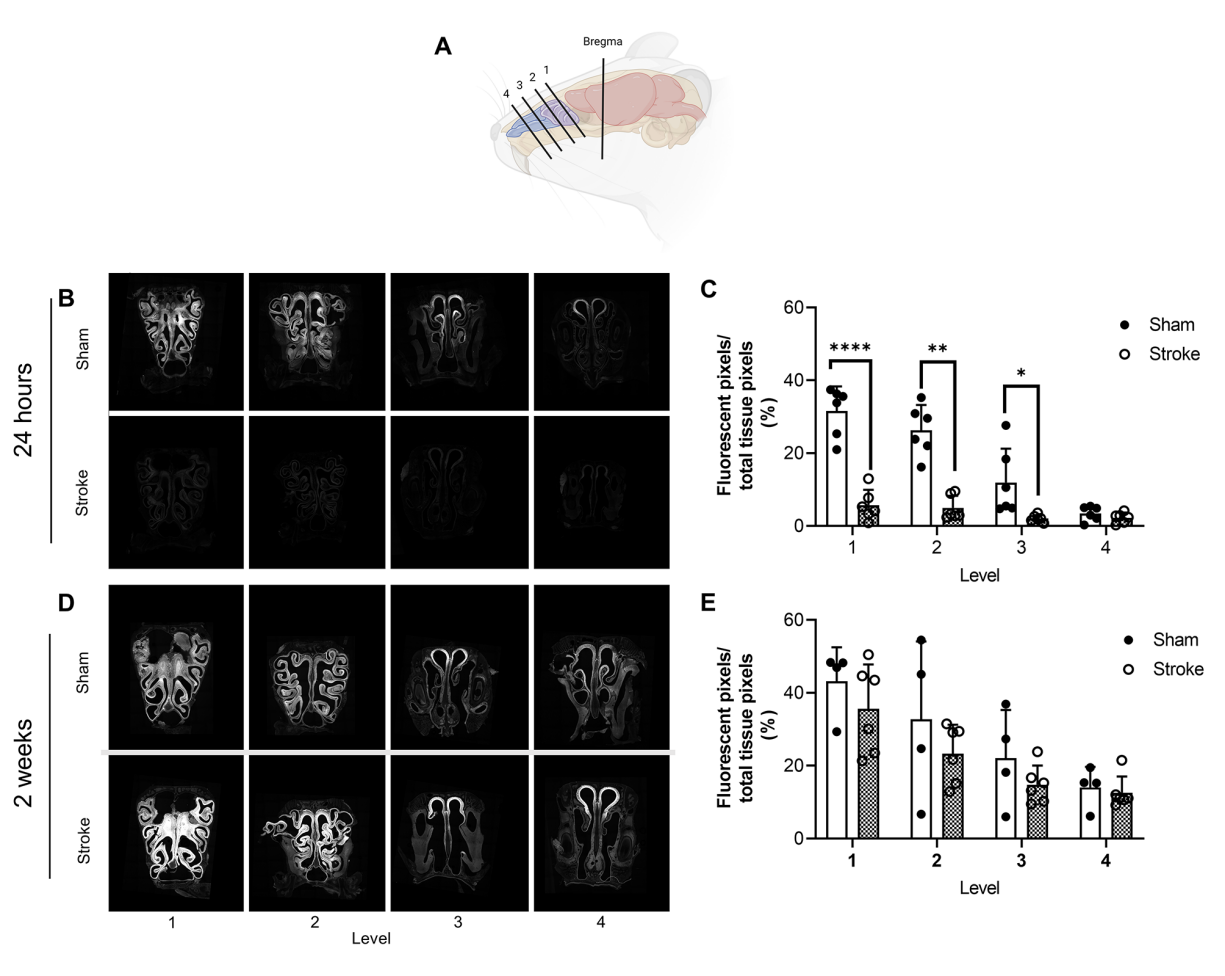
CSF tracer efflux into the nasal mucosa via the cribriform plate. (A) Schematic indicating the four levels of sections taken from the mouse nose. (B) Representative images of fluorescent CSF tracer distribution and load in the nasal mucosa 24 h after photothrombotic stroke. (C) Fluorescent tracer load as a percent of total tissue area at each of the four levels 24 h after stroke. (D) Representative images of fluorescent CSF tracer distribution and load in the nasal mucosa two weeks after photothrombotic stroke. (E) Fluorescent tracer load as a percent of total tissue area at each of the four levels 2 weeks after photothrombotic stroke (E). **** p < 0.001, ** p < 0.01, *p < 0.05. Schematic created with BioRender.com

Similar patterns of CSF tracer load were noted in the ipsilateral and contralateral stroke hemispheres (Fig. 3). Significant differences were seen between dorsal and ventral regions in both hemispheres at both time points. There was also significantly more tracer in the ventral region compared to the lateral region of the ipsilateral (stroke) hemisphere at 24 h and 2-weeks as well as significantly more tracer in the lateral region compared to the dorsal region in both hemispheres at 2-weeks post-stroke.

### CSF outflow via the nasal mucosa

At 24 h, there was significantly less tracer in nasal mucosa of stroke animals compared to sham. This was seen at all levels except the most distal to the cribriform plate (level 4), at which there was very little tracer detected in either group (level 1: stroke = 5.72% ±4.21, sham = 36.1% ±6.77, p < 0.0001; level 2: stroke = 4.96% ±3.32, sham = 26.3% ±6.92, p = 0.0022; Level 3: stroke = 2.07% ±0.97, sham = 11.9% ±9.30, p = 0.0481; Fig. 4b and c). At 2 weeks after stroke/sham surgery there were no significant differences between stroke and sham at any of the examined levels (Fig. 4d and e).

## Discussion

In this study we have shown that at 24 h post-stroke, there is a reduction in both the influx of CSF-born tracer into the brain parenchyma and efflux to the nasal mucosa. However, these changes in CSF dynamics were not present two weeks after stroke.

The reduction in CSF tracer load in the brain was present in the dorsal region of both the ipsilateral and contralateral hemisphere of stroke animals. The mean values in the lateral and ventral regions were in the same direction, lending credence to this finding. This indicates a global impairment of CSF circulation at 24 h post stroke.

A global decrease in CSF circulation has previously been reported by Wang et al. (2017) in a microinfarct model at three days after injury in both adult and aged mice [9]. This model generated microinfarcts by embolization of cholesterol crystals from the common carotid artery, but it is not stated whether embolization via the anterior communicating artery was excluded which is a possible explanation for the global decrease in CSF circulation in their model. Wang et al. also found that global impairment of CSF circulation was restored two weeks after stroke, which aligns with our findings in this study. Gaberel et al. (2014) reported reduced CSF tracer load in the ipsilateral hemisphere in an embolic clot middle cerebral artery occlusion stroke model at three hours post stroke [8]. This deficit in CSF dynamics had resolved by 24 h, when spontaneous vessel recanalization had occurred in all animals. It is possible that Gabarel et al. did not detect a global decrease in CSF tracer movement due to comparing CSF tracer load in the ipsilateral hemisphere to the contralateral hemisphere rather than sham brain tissue. Howe et al. (2019) reported reduced CSF tracer load in the ipsilateral hemisphere 30 days after stroke [10], but again, this study compared CSF tracer influx in the ipsilateral hemisphere to that of the contralateral, missing any potential global disturbance in CSF movement. This extended timeline for the disruption of CSF dynamics has not been supported by any other studies. Arbel-Ornath et al. (2013) reported a reduction in CSF movement immediately following photothrombotic occlusion of a pial artery [24] but did not examine CSF tracer load at sites distal to the injury or at other time points. While not measured in this study, the results of Arbel-Ornath et al. and Gabarel et al. may be explained by reduced arterial pulsatility, given that the cardiac cycle and arterial pulsatility are important in CSF circulation [6, 27–29]. Such a mechanism may have contributed to the reduced intensity of CSF tracer observed in the dorsal region in our study due to its proximity to the lesion. This does not, however, explain changes in brain regions distal to the injury site, particularly the contralateral hemisphere of stroke animals. These global changes also suggest edema is unlikely to have played a large role.

A potential mechanism for the results seen in this study is cortical spreading depolarisation (CSD) induced closure of perivascular pathways. CSDs (also known as peri-infarct depolarisations in stroke-specific contexts) are a well-recognised post-stroke phenomenon of a wave of depolarisation spreading across the cortex extending from the infarct to neighbouring tissue. CSDs can extend beyond the initial ischaemic territory, altering vascular dynamics in previously uninjured tissue, and may occur numerous times following an ischaemic insult, including following photothrombotic stroke [30]. Schain et al. (2017) showed, in mice, that a single artificially induced CSD caused almost complete closure of the perivascular space of surface and penetrating arterioles for 30 min [31]. They also showed that this resulted in reduced clearance of intraparenchymally injected tracer. At present, there are no definitive reports of how long after stroke CSDs persist, but there is evidence to indicate that CSDs are unlikely to be present two weeks after stroke [32]. Based on these reports, it is possible that repeated CSDs, and therefore repeated closure of the perivascular spaces, as well as alterations to the neuronal microenvironment caused by such events, could contribute to disruption of CSF dynamics distal from the stroke site as observed in the lateral region of the contralateral hemisphere in this study.

The mechanisms of fluid transport in the brain parenchyma are not clearly defined and there is evidence that it is driven by diffusion, convection or a combination of the two. Cserr et al. (1981) found that radiolabelled tracers of varying molecular weights injected intraparenchymally were cleared from the brain at a similar rate, indicative of convective transport [33]. Koundal et al. (2020) developed an *in silico* model of parenchymal fluid movement based on CSF tracer movement *in vivo* in rats. Only when both convective and diffusive mechanisms were considered could the model recapitulate the *in vivo* flow [34]. However, Smith et al. (2017) found that diffusive transport was the dominant mechanism in their study when fluorescent tracers of varying molecular weights were cleared in a size-dependent manner [35]. No matter the mechanisms driving CSF/ISF movement in the parenchyma, it must move through the extracellular space (ECS). A reduction in the volume of the ECS may account for the reduction in tracer load observed in this study. Thorne and Nicholson (2006) found that terminal ischemia results in a dramatic decrease in ECS volume from ~ 20% to ~ 5% of brain volume. [36]. Other neurological insults have been shown to reduce the diffusion of dextran tracers in the parenchyma [37]. Conceivably, a narrowing of the ECS could result in an overall reduction in tracer load within the tissue and contribute to the results seen here.

Interestingly, there were significant differences in tracer load in different brain regions in sham animals at both 24 h and two weeks, with the highest load present in ventral tissue followed by lateral then dorsal tissue. This indicates that the flow of tracer following cisterna magna injection preferences entry into the parenchyma from the ventral region with progressively less in the lateral and dorsal brain regions. This pattern has also been observed by both Proescholdt et al. (2000) and Lee et al. (2015) in rodents in the prone position following cisterna magna injection [26, 38]. Stroke brains were more variable in which regions were significantly different from each other, however, the ventral region consistently had significantly higher tracer load compared to the dorsal region at both time points and in both the ipsilateral and contralateral hemispheres. This result may speak to the pattern of CSF flow in rodents and be an important consideration for CSF-related experiments using intracisternal tracer injection.

In this study we report an almost complete halting of CSF tracer outflow from the cranial space via the cribriform plate at 24 h after stroke. Our findings suggest that our previously observed reduction in CSF drainage to the deep cervical lymph nodes after stroke [16] may be due to reduced CSF outflow via the olfactory route, at least in rodents. Previous studies have extensively demonstrated that the cribriform plate is a major site of CSF outflow. The long-standing dictum that CSF exits the cranial space via the arachnoid granulations where it drains into the superior sagittal sinus [39] ignores early work by Schwalbe who demonstrated that Berlin blue injected into the subarachnoid space migrated to the cervical lymphatic system in animals [40]. In addition to this, work by Zwillinger (1912) demonstrated that a tracer injected into a human infant drained into the nasal mucosa [41]. Despite this evidence, the importance of the cribriform plate as a key site of CSF efflux has been dismissed by some groups, especially in bipedal animals with a less prominent olfactory bulb, such as humans. To examine this Johnston et al. (2004) injected a brightly coloured silicone rubber compound (Microfil) into the subarachnoid space of post-mortem tissues from several species, including a human cadaver. In all species examined they found convincing evidence of nasal-lymph connections, but variability in tissue freshness resulted in different patterns of Microfil distribution that negated attempts to compare CSF outflow between species [20]. Koh et al., (2005) further highlighted the importance of cribriform plate lymphatics as a site of CSF/ISF drainage by listing the extensive number of published studies that demonstrated drainage of CSF tracers to the retropharyngeal cervical lymph nodes via the olfactory nerves and nasal lymphatics [42]. Given the importance of CSF drainage via the cribriform plate, our findings indicate that reduced CSF outflow via the olfactory route after stroke may contribute to our previously identified ICP elevation. Indeed, a study by Silver et al. (2002) found that blocking the cribriform plate of sheep with dental wax reduced cranial compliance during infusion of artificial CSF, leading to an increase in ICP [43].

While the cribriform plate is a key site of CSF outflow, there are other sites of outflow such as the spinal and cranial subarachnoid granulations and nerve roots, and meningeal lymphatics which should serve as compensatory outflow sites to help maintain cranial compliance. Given our findings of reduced CSF outflow via a key lymphatic drainage route in this paper, it is also possible that other routes of drainage of CSF to lymph nodes, such as via foramina in the skull [17], is reduced after stroke but further work is required to establish this. Work by our team has identified an inverse correlation between ICP elevation and CSF tracer transit time to the spinal subarachnoid space, indicating that ICP elevation was associated with faster CSF tracer transit from the cranial space to the spinal subarachnoid space [16]. It remains unclear whether reduced transit time to the spinal subarachnoid space indicates increased outflow at spinal subarachnoid granulations and nerve roots, or greater intraspinal circulation. Furthermore, it is possible that the increased pace of CSF transit to the spinal subarachnoid space means that the intracisternally injected CSF tracer in our present study migrates to the spinal subarachnoid space due to a mechanism preventing it migrating into the cranium and thus into the brain and subsequently the nasal mucosa. This could explain the reduced tracer load in both brain and nasal mucosa observed in this study. Another possibility is altered CSF absorption from the subarachnoid space into meningeal lymphatic vessels. Meningeal lymphatic vessels were described in mice by Aspelund et al. (2015) and Louveau et al. (2015) as an important ‘bridge’ for the clearance of CSF to sites of outflow, such as the nasal mucosa and cervical lymph nodes [44, 45]. A study by Pu et al. (2019) found that following subarachnoid hemorrhage in mice, there was a decrease in tracer load in deep cervical lymph nodes (the assessed site of CSF outflow) coupled with a reduced volume of tracer in brain tissue [46]. This mirrors the results of the current study where a decrease in both brain and outflow site tracer load was observed. In their study, Pu et al. noted that intracisternally infused tracer appeared to have collected in the meninges. Examination of the meninges following photothrombotic stroke may shed light on the role of the dural lymphatics in the results seen here.

In this study we used a photothrombotic stroke model in mice to examine how CSF movement is altered after stroke. We elected to examine the CSF system at 24 h after stroke to determine whether disruptions occur when ICP elevation is known to peak in our previous rat studies. Furthermore, we opted to use the photothrombotic model of stroke as it produces a focal lesion, better allowing us to interrogate whether global disturbances in CSF dynamics occur after stroke and allowing us to rule out arterial pulsatility as the sole contributor to changes in CSF tracer load. A strength of this study was the use of ketamine/xylazine anesthetic. Performing experiments such as those included in this study in awake or naturally sleeping mice is difficult, therefore we chose an anesthetic regimen that most closely mimics normal physiology [22] as opposed to inhalation anesthetics that have been shown to significantly alter the CSF system [47].

One limitation of this study is that physiological variables known to be important in CSF dynamics, such as heart rate and respiration, were not assessed. While potentially important to have such data, this approach was taken to reduce the number of invasive procedures performed that could potentially alter physiology and influence results. Similarly, while a primary motivation in undertaking the study was to try to understand the mechanism for our previously reported increased ICP and increased CSF outflow resistance at 24 h post-stroke, it was not possible to measure these parameters without compromising the integrity of the CSF tracer diffusion data.

## Conclusions

This study identifies global alterations to CSF movement that results in reduced CSF tracer influx into the parenchyma and reduced outflow via the cribriform plate at 24 h after stroke. This provides insights into the potential mechanism, disrupted CSF dynamics, contributing to ICP elevation after stroke and highlights promising avenues for future research in both stroke and other conditions where CSF dynamics are disturbed. It will be important for future studies to tease apart the mechanisms driving altered CSF outflow in order to better treat stroke patients and others with pathological alterations to CSF dynamics.

## Electronic supplementary material

Below is the link to the electronic supplementary material.

Supplementary Material 1
Time course of intracisternal injection of Texas Red Dextran 3 kDa load in brain tissue from na?ve animal.

Supplementary Material 2
Pixel intensity over tissue depth of all included animals at 24 hours (left) and 2 weeks (right) post-stroke for the dorsal (top), lateral (middle) and ventral (bottom) brain regions. Individual animal values are shown by the grey lines and means for ipsilateral stroke, contralateral stroke and sham animals are plotted in colour

## Data Availability

All data generated or analysed during this study are available from the corresponding author.

## Abbreviations

CSD: Cortical spreading depolarisation
CSF: Cerebrospinal fluid
ICP: Intracranial pressure
ISF: Interstitial fluid
ROI: Region of interest
SD: Standard deviation
