# Supplementary figures and images for: Movement of cerebrospinal fluid tracer into brain parenchyma and outflow to nasal mucosa is reduced at 24 h but not 2 weeks post-stroke in mice
Source: Fluids Barriers CNS. 2023 Apr 11;20:27. doi: 10.1186/s12987-023-00427-2 (PMC10088200; doi:10.1186/s12987-023-00427-2)

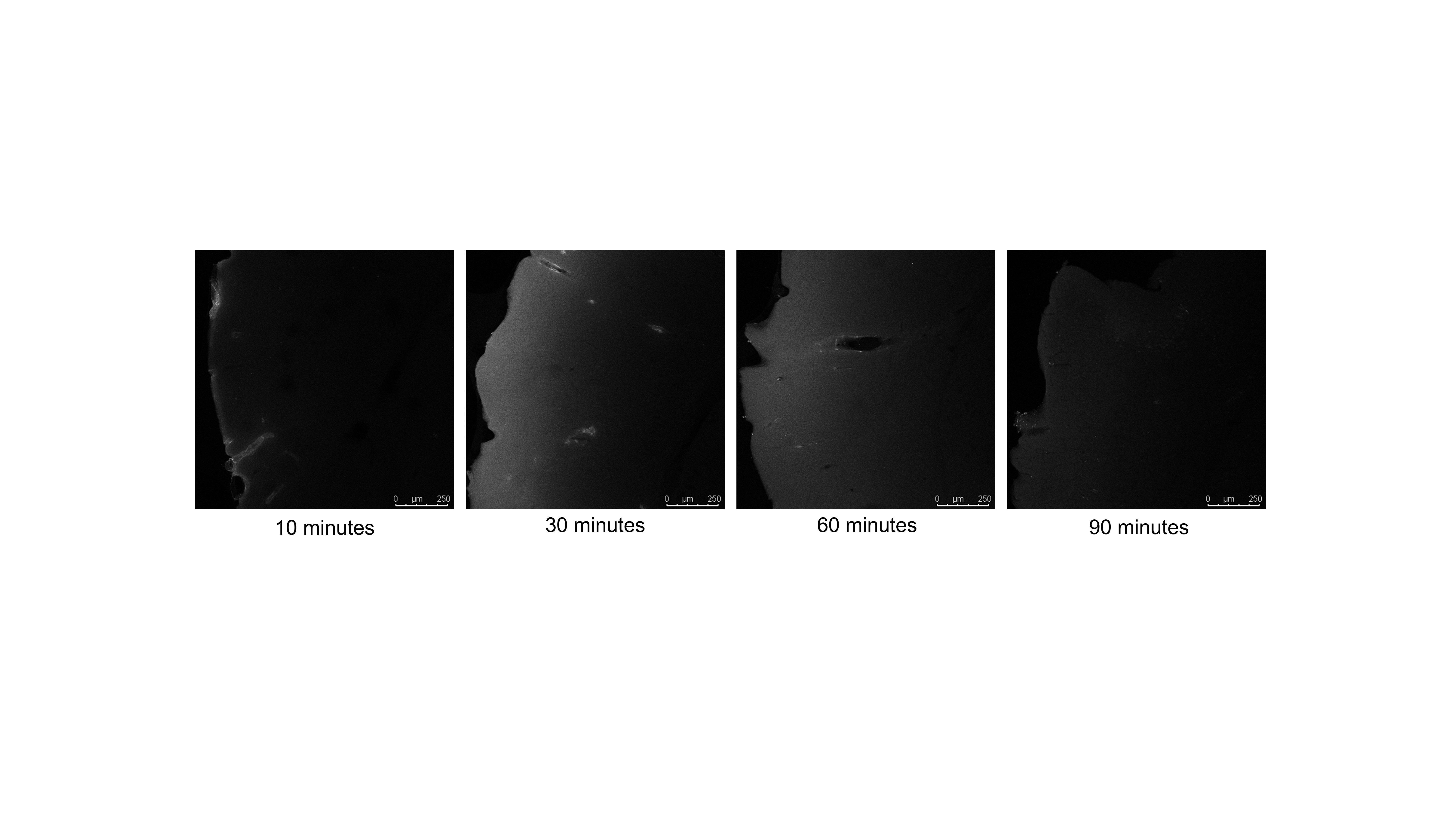

Supplement: Supplementary file 1 — Supplementary Material 1 Time course of intracisternal injection of Texas Red Dextran 3 kDa load in brain tissue from na?ve animal. [file 12987_2023_427_MOESM1_ESM.tif]

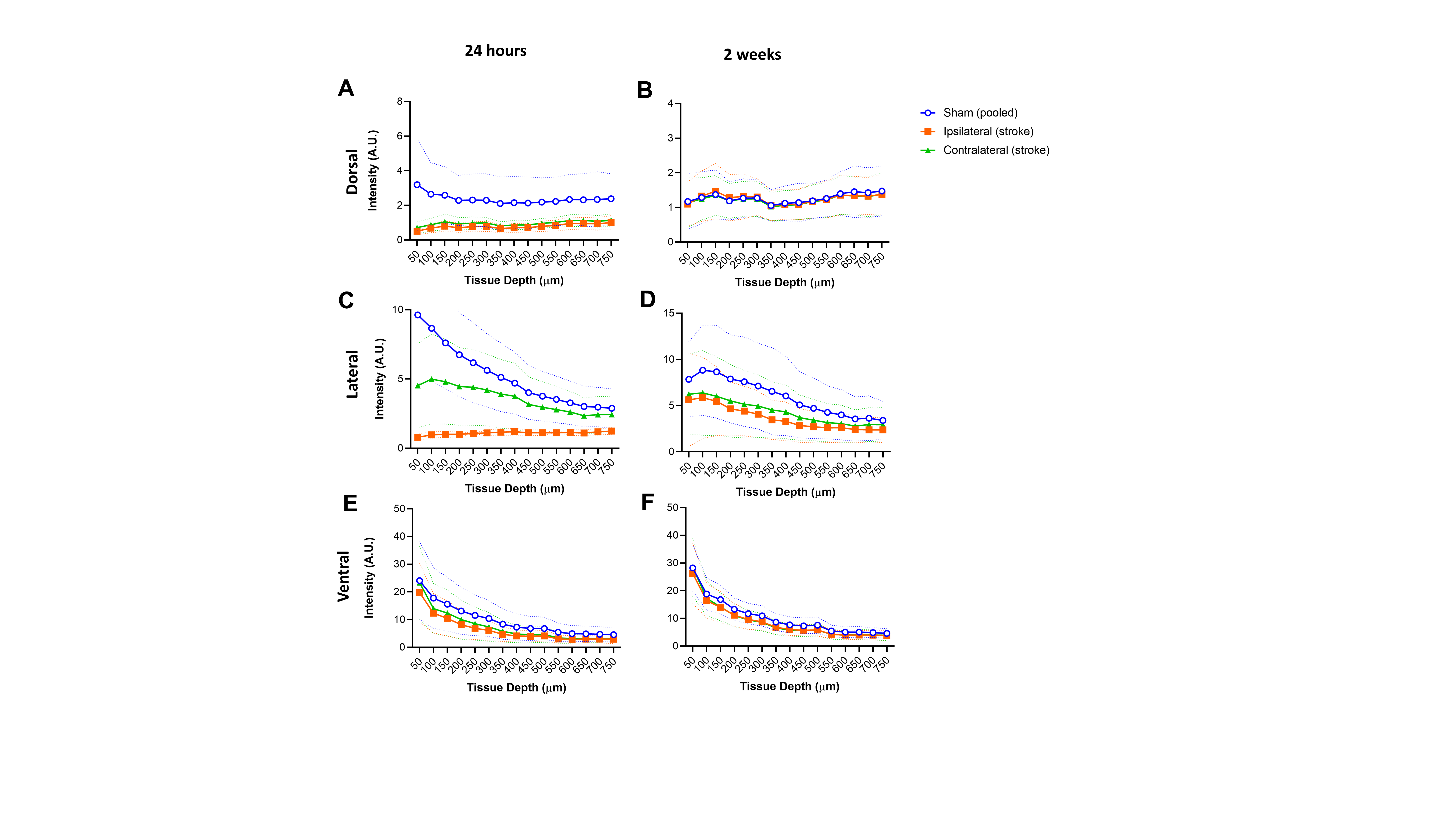

Supplement: Supplementary file 2 — Supplementary Material 2 Pixel intensity over tissue depth of all included animals at 24 hours (left) and 2 weeks (right) post-stroke for the dorsal (top), lateral (middle) and ventral (bottom) brain regions. Individual animal values are shown by the grey lines and means for ipsilateral stroke, contralateral stroke and sham animals are plotted in colour [file 12987_2023_427_MOESM2_ESM.tif]
